# Heat stress and soil thermal gradients shape root-associated fungal community recruitment

**DOI:** 10.3389/fmicb.2025.1334648

**Published:** 2025-08-12

**Authors:** Pablo Catarecha, Eoghan King, Sandra Díaz-González, Elena Caro, Soledad Sacristán, Juan Carlos del Pozo

**Affiliations:** ^1^Centro de Biotecnología y Genómica de Plantas (UPM-INIA/CSIC), Universidad Politécnica de Madrid (UPM) - Instituto Nacional de Investigación y Tecnología Agraria y Alimentaria-CSIC (INIA/CSIC), Campus Montegancedo, Madrid, Spain; ^2^Departamento de Biotecnología-Biología Vegetal, Escuela Técnica Superior de Ingeniería Agronómica, Alimentaria y de Biosistemas, Universidad Politécnica de Madrid (UPM), Madrid, Spain

**Keywords:** abiotic stress, fungal community, temperature, *Solanum lycopersicum*, metagenomics, root, climate change

## Abstract

Climate change is increasing the overall temperature of the planet and increasing the number of extreme heat waves events. These phenomena are negatively affecting crop production and food security. Thus, under this scenario, understanding the adaptations that encompass the plant response to high temperature will be essential to enhance crop tolerance and yield. Plant responses to elevated temperature rely on both genetic factors and the dynamic interplay with the surrounding microbiota. Recently, the role of root microbiota as a key player in the plant’s response to heat, is gaining significant relevance. This work presents the analysis of fungal microbiota from the rhizosphere and the root-associated fractions of tomato roots in response to high temperature. Although the analyses were done in an enclosed environment, we used the TGRooZ (Temperature Gradient Root Zone) system to mimic field conditions. The TGRooZ generates a temperature gradient like the natural soil during a heat wave event. We found that distinct soil/root compartments assemble a different fungal community, with the rhizosphere fraction exhibiting greater diversity and abundance, while the root-associated fraction was enriched in fewer but more specialized taxa. Notably, the experimental conditions used to analyze heat responses significantly influenced the final microbiome composition. Our data suggest that the TGRooZ system will enable more accurate analysis of plant-microbiome responses to heat stress and help evaluate the potential of beneficial microbes to enhance crop productivity under near-natural conditions.

## 1 Introduction

Climate change is driving a rise in global temperatures over the coming decades (Zandalinas et al., 2021; IPCC, 2023; Abebaw, 2025) and increasing the frequency and intensity of extreme heatwaves (Maraun et al., 2025). These changes are having a detrimental impact on crop yields and threatening global food security. These extreme events threaten to trigger unprecedented declines in crop yields, severe economic losses and threatening the food security (Miller et al., 2021). A recent study has described how global warming will impact the growth, the development and the productivity of both wild plants and crops worldwide (Bhattacharya, 2019), pushing agricultural ecosystems to adapt to increasingly harsh conditions (Fahad et al., 2017). Given these challenges, two critical questions have raised: “to what” *extent will climate change reduce crop yields?* and *How can biotechnological innovations mitigate these impacts?* These concerns are now top priorities for researchers, governments, and society (Gray and Brady, 2016; Calleja-Cabrera et al., 2020, Abebaw, 2025).

When exposed to temperatures above their optimal range (warming), plants undergo a series of phenotypic changes to adapt their growth and development to the new conditions. However, prolonged exposure to warming temperatures often reduces vegetative growth, seed production, and overall yield (Giri et al., 2017). The adaptive responses to warming, termed thermomorphogenesis (Quint et al., 2016), involve modifications in growth patterns that help plants acclimate to moderate temperature increases. Thermomorphogenesis integrates multiple physiological and developmental processes, such as hypocotyl elongation, root growth, and flowering, through hormone signaling, protein modifications, chromatin remodeling, and changes in gene expression to conform a network of developmental, molecular, and genetic adaptations (Zhao et al., 2020; Lee et al., 2021). Temperature sensing, which initiates the thermomorphogenic responses, is primarily mediated by phytochrome signaling in leaves (Lee et al., 2021) and shoot-to-root communication involving *HY5* activity and auxin transport (Gaillochet et al., 2020). In addition to phytochromes, other mechanisms, such as protein and RNA stability, membrane fluidity, and protein folding, contribute to temperature perception across different plant tissues (Kerbler and Wigge, 2023).

Opposite to warming, extreme temperature events (heat stress) can severely disrupt plant growth and development (González-García et al., 2023) and induces adaptive memory in the affected tissues (Perrella et al., 2022). Heat stress predominantly impacts the aerial plant tissues due to direct exposure to high atmospheric temperatures, whereas roots experience milder effects. This differential response can be explained by the soil’s geothermal properties, which creates a temperature gradient that decreases with depth (González-García et al., 2023). Therefore, this soil temperature gradient helps protect the root system and surrounding microbiome from the detrimental effects of excessive heat. In recent years, the critical role of the root system in promoting plant adaptation to various stresses, including heat stress, is becoming increasingly evident. Roots are vital for maintaining water and nutrient homeostasis (Cochavi et al., 2020; González-García et al., 2023) and for facilitating interactions with the soil microbiome, which supports essential physiological processes and stress responses (Chaparro et al., 2012; Liu et al., 2020). Plant response to heat stress, however, does not only involve alterations in the plant’s physiology or developmental processes. The root-soil microbiota plays a critical role to support essential plant physiological processes and in the adaptation to both biotic and abiotic stress in a climate change scenario (Chaparro et al., 2012; Liu et al., 2020; Zeng et al., 2025). Different researches have demonstrated that plant roots engage in dynamic communication with the soil microbiome (Enagbonma et al., 2023), actively recruiting beneficial microbes that enhance root exploration for proper development and mitigate the effects of nutrient deficiency, water scarcity, and other stress conditions (Finkel et al., 2019; de la Fuente Cantó et al., 2020; Molefe et al., 2023; Liu et al., 2020). Prolonged high temperatures modify soil microbial diversity and favor the establishment of specific root-microbiota interactions (Nottingham et al., 2019; Sharma et al., 2023). Despite intense research on the adaptation of root microbial communities in response to heat stress, there are two main aspects that have been overlooked. First, to date, much of the research on heat stress and heat tolerance in plants has been performed *in vitro* or greenhouse experiments, where plants (roots and shoot) were uniformly heated, or root-cooled systems (Heckathorn et al., 2013; Prerostova et al., 2021). However, natural soils forms a decreasing temperature gradient from the top surface to deeper layers, buffering the excessive high temperature during extreme heat waves and seasonal variations (Lynch et al., 2012; Brunetti et al., 2022). Recently, a novel device, called TGRooZ, is capable of generating a temperature gradient in soil-filled pots or agar-based *in vitro* plates, while simultaneously heating the aerial parts. The use of the TGRooZ has enabled the study of heat stress in enclosed. The environments but in a near-natural conditions (González-García et al., 2023). That work demonstrates the negative, but rather artifactual, effect of high temperature on root development and responses to heat as well as the effect on bacterial association and the bias introduced when homogeneous temperature in shoot and root is used. The second factor introducing bias into heat-related root microbiome research is the predominant focus on bacterial communities over fungal ones. Although there are some works indicating the role of arbuscular mycorrhizal fungi to increase tolerance to the combination of heat and drought stresses in tomato (Duc et al., 2018) or recent reviews highlighting the importance of the fungal microbiome (Fontana et al., 2021; Pozo et al., 2021), the number of studies of fungal communities in the context of heat stress are rather low. Thus, studying the effect of heat stress on fungi communities and their relationship with plants as well as the identification of new fungi that enhance plant growth and resilience to temperature increments is needed.

This study investigates the assembly of fungal communities in the rhizosphere and root-associated microbiome of tomato plants grown under heat stress in near natural conditions. We compared fungal community composition between plants grown under uniform temperature conditions (both roots and shoots exposed to equal heat or optimal temperatures) and plants subjected to shoot heating while maintaining their roots in a temperature gradient using the TGRooZ device, which better mimics natural soil conditions. Our results demonstrate that excessive temperature in the soil-root ecosystem significantly alters fungal communities in both rhizospheric and root-associated fractions. These findings highlight the importance of using systems like the TGRooZ, which prevents root overheating while establishing a natural temperature gradient, to obtain reliable data on plant-microbiome interactions during heat stress studies.

## 2 Materials and methods

### 2.1 Plant material, DNA extraction and sequencing

Tomato plants variety “Moneymaker” were planted in pots using natural soil taken from a the Polytechnical University of Madrid-Campus of Montegancedo (coordinates 40.4055923, −3.8308637) that was mixed with clean river sand in a proportion 3:1 as published earlier (González-García et al., 2023). Briefly, tomato plants were germinated for 10 days in vermiculite and then transferred to 3.5-liter pots containing the soil:sand mix. To mimic close to natural conditions, we used the TGRooZ (Temperature Gradient in the Root Zone) device (González-García et al., 2023). The TGRooZ device generates a temperature gradient based on the differential temperature between the top atmospheric (36°C) and the base of the device (7°C), generating a temperature gradient in the soil of the pots that range from 36°C (topsoil layer) to 24°C (deeper soil layer), like the conditions found in natural soils during a high temperature period.

**Figure 1:**
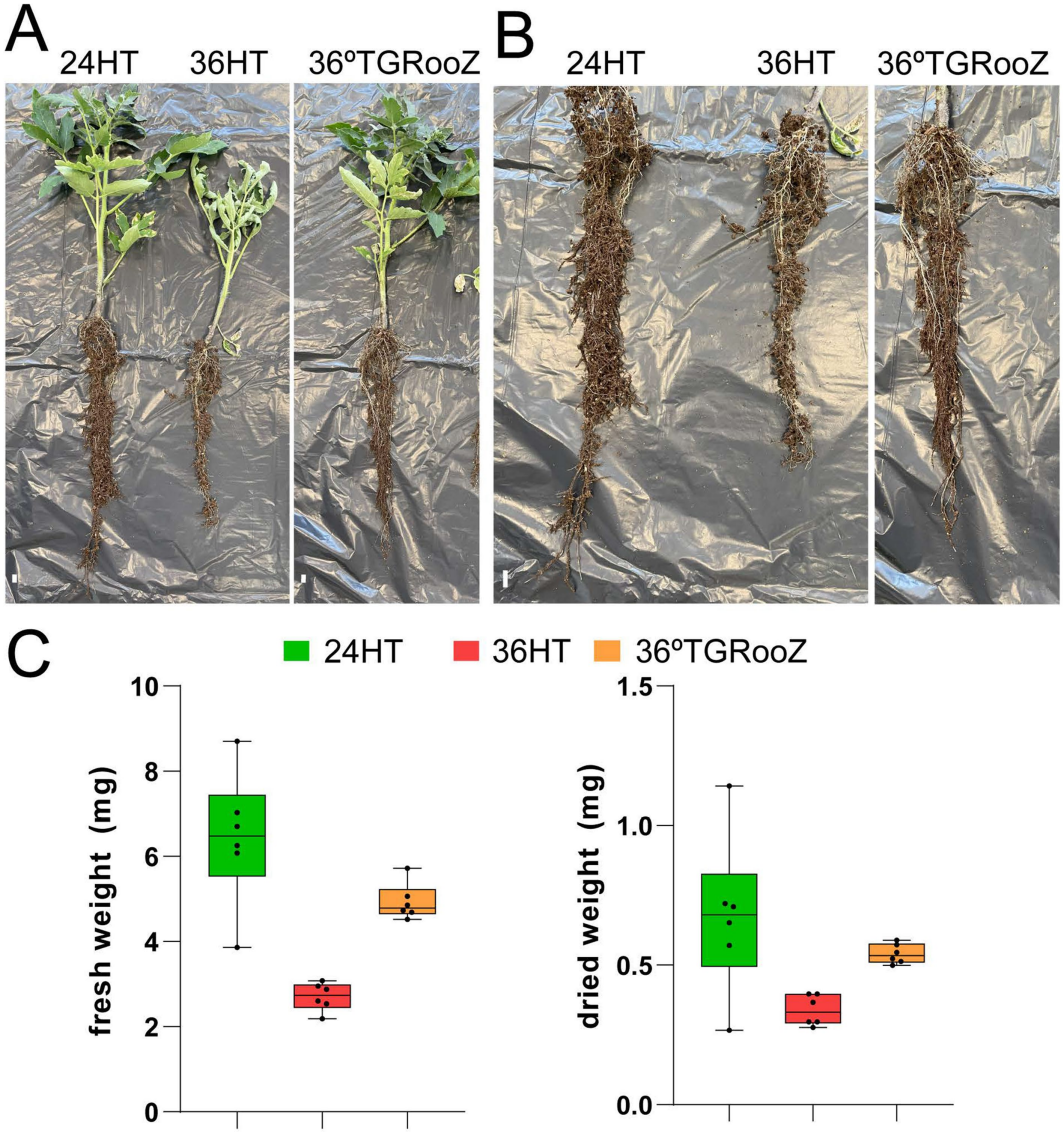
Heat stress affects tomato plant development. **(A)** Tomato plants were grown for 3 weeks in soil containing pots in an enclosed growth chamber at 24°C homogeneous in root and shoot (24H), at 36°C homogeneous in root and shoot (36H) or at 36°C in the shoot and in a temperature gradient in the root (36TGRooZ). Afterwards, the root system was taken out of the pot, conserving the attached soil that was used to purify the rhizosphere fraction. **(B)** Higher magnification of the root system from plants shown in A. White bars correspond to 1 cm (left panel) or 3 cm (right panel). **(C)** Fresh and dried weight of the aerial part of plants grown as in **(A)**.

Tomato seedlings were grown in three different temperature conditions: uniform temperature of 24°C for shoot and roots (24HT), uniform temperature of 36°C for shoot and roots (36HT) or 36°C in the shoot and a gradient for the roots (36TGRooZ). After 3 weeks, we harvested the rhizospheric fraction and the root-associated fraction of the roots. For the rhizospheric fraction, the soil attached to the roots (Figure 1) were first briefly rinsed (5 s) with 1 L of sterile water. Then, the rhizosphere fraction (soil tightly adhering to the roots) for each sample was collected by vigorously shaking the roots in 20 mL of sterile water. The soil, without roots, was then decantated by centrifugation (5 min at 3,000 rpm) and frozen for further analysis. To harvest the root-associated fraction, roots were washed twice with 100 mL of sterile water until they appeared clean and then the roots were frozen. Notice that no surface sterilization was performed. We harvested and analyzed six independent replicates for each condition. Additionally, a sample of five replicates of bulk soil was taken before plantation to analyze the initial fungi community.

All the samples were sent to BiomeMakers^1^ in Sacramento, United States. The DNA was extracted from each fraction, along with the original bulk soil and after quality check it was used for PCR amplification following the Illumina’s two-step PCR protocol. Libraries were prepared for the ITS1 region using Biome Makers custom primers (Becares and Fernandez, 2016) and subject to Illumina MiSeq using paired-end sequencing (2 × 300 bp).

### 2.2 Fungal ITS metabarcode pipeline

The computational analysis on ITS metabarcode data was performed using the DADA2 pipeline (Callahan et al., 2016) in the Qiime2 environment (Bolyen et al., 2019). Raw fastq data was imported into the Qiime2 platform using standard plugins and read quality from all samples was analyzed with q2-demux summarize. Reads were then truncated with AUTOTRUNC,^2^ an in-house developed script designed to pick the truncation point at the length where per-base median quality falls below average quality, which is roughly the point where the quality shows the maximum decreasing slope. AUTOTRUNC relies on a headless FastQC (Andrews, 2010) analysis on every fastq file included in the study, and detects the point where the median PHRED score drops below the average quality on a per-base basis. This position is stored as the truncation point for each read. The script then calculates the average truncation point for forward and reverse reads, limiting the truncation of forward reads whenever the expected overlap with reverse reads is lower than the default overlap of 12 nt needed for DADA2. This limitation only affects the forward reads to take advantage of the expected higher quality of these, compared to reverse reads, whose quality is likely to be lower. The truncation points are stored as script variables $R1TRUN and $R2TRUN and passed to DADA2 when needed.

Imported reads were denoised and demultiplexed with q2-dada2 using the following parameters: --p-trunc-len-f $R1TRUN --p-trunc-len-r $R2TRUN --p-trim-left-f 5 --p-trim-left-r 5 --p-pooling-method pseudo. Due to the low quality of the reverse reads, both single-end and paired-end approaches were tested to determine which provided more accurate results. The single-end approach used only the forward reads after trimming and AUTOTRUNC processing. This strategy retained approximately 49% of the initial reads and yielded nearly twice the number of ASVs compared to the paired-end approach (Table 1). Therefore, all subsequent analyses were performed using single-end data based on forward reads only. Visualizers for denoised representative sequence data were created using q2-feature-table tabulate-seqs (Johnson et al., 2008; NCBI Resource Coordinators, 2017). The resulting Amplicon Sequence Variant (ASV) table was filtered for low-frequency ASVs using q2-feature-table filter-features with the parameters --p-min-frequency 100 --p-min-samples 3.

**Table 1:** DADA2 denoising stats.

	Input	Filtered	% filtered	Denoised	Merged	% merged	Non-chimeric	% non-chimeric
Paired reads	187,027,32	81,053,93	43.34%	79,307,12	73,786,66	39.45%	52,885,46	28.28%
Single reads	187,027,32	128,133,12	68.51%	125,960,78			91,467,12	48.91%

Phylogenetic analyses on ASVs were performed using q2-phylogeny align-to-tree-mafft-fasttree (Katoh and Standley, 2013; Bolyen et al., 2019). Taxonomic classification of ASVs was performed with the plugin q2-feature-classifier (Bokulich et al., 2018) using the UNITE ITS databases (Nilsson et al., 2019; Kõljalg et al., 2020). A naive Bayes classifier was trained on ITS data with the method fit-classifier-naive-bayes, which was subsequently used to classify ASVs with the method classify-sklearn (Pedregosa et al., 2011). This was performed both with eukaryotic and fungal ITS data using the dynamic version of the UNITE database. A preliminary taxonomical analysis was performed using the eukaryotic ITS database from UNITE, in order to detect and filter out non-fungal ASVs that could contaminate downstream analysis. Taxonomic metadata derived from the eukaryotic classifier was used to keep fungal ASVs, along with unclassified features using q2-taxa filter-seqs and q2-taxa filter-table with the parameter --p-include k__Fungi, Unassigned. The filtered sequences and features were then re-classified using the fungal-only ITS database from UNITE.

Alpha and beta diversity of fungal communities were analyzed using q2-diversity core-metrics-phylogenetic, which performs a standard series of analysis that outputs a set of common distance matrices and visualization tools, including Principal Coordinates Analysis (PCoA) plots (Halko et al., 2011; Legendre and Legendre, 2012). Alpha-diversity (diversity within samples) analysis was carried out based on the number of observed ASVs (richness) and Shannon index. Non-paramentric Kruskal-Wallis test (*p* < 0.05) was conducted using alpha-group-significance. Beta-diversity analysis was carried out by calculating the unweighted UniFrac distance. Permutational multivariate analyses of variance (PERMANOVA) were performed using the pairwise beta-group-significance (Anderson, 2001) from plugin q2-diversity.

PCA metrics were calculated with GEMELLI (Martino et al., 2019), using method phylogenetic-rpca-with-taxonomy. This plugin performs robust Principal Component Analysis (rPCA) using Aitchison distance matrices on sparse data (Martino et al., 2019). Differentials were calculated with SONGBIRD (Morton et al., 2019) using the method multinomial with the following parameters: --p-formula “C(Temperature, Treatment(‘24HT’))” --p-min-sample-count 1 --p-min-feature-count 1 --p-summary-interval 1 --p-epochs 10000. Minimum sample and feature counts were set to 1 as the input for q2-songbird was already filtered data; the distances were calculated relative to the reference temperature condition ‘uniform 24HT’, as this is considered to be the standard testing temperature when not using TGRooZ.

Computational analysis of fungal composition was performed using two different approaches: Analysis of Compositions of Microbiomes (ANCOM) (Mandal et al., 2015) and Anova Like Differential Expresion (ALDEx2) analysis (Fernandes et al., 2013; Gloor et al., 2016), using the visualization tools provided therein. q2-composition ancom was used to detect differentially abundant ASVs across all conditions, while q2-aldex2 aldex2 was used to perform pairwise comparisons for all three pairs of temperature conditions. Visualization of compositional plots and biplots (Aitchison and Greenacre, 2002) was performed with EMPEROR (Vázquez-Baeza et al., 2017). Differentials were visualized using QURRO (Fedarko et al., 2020). Heatmaps were made using q2-feature-table heatmap, which is a wrapper for MATPLOTLIB heatmaps (Hunter, 2007). Feature lists were filtered with VENNY (Oliveros, 2007). Computationally demanding analyses were performed at the computational cluster of the Centro de Biotecnología y Genómica de Plantas (CBGP, UPM-INIA/CSIC).

## 3 Results

### 3.1 Taxonomical profile

**Figure 2:**
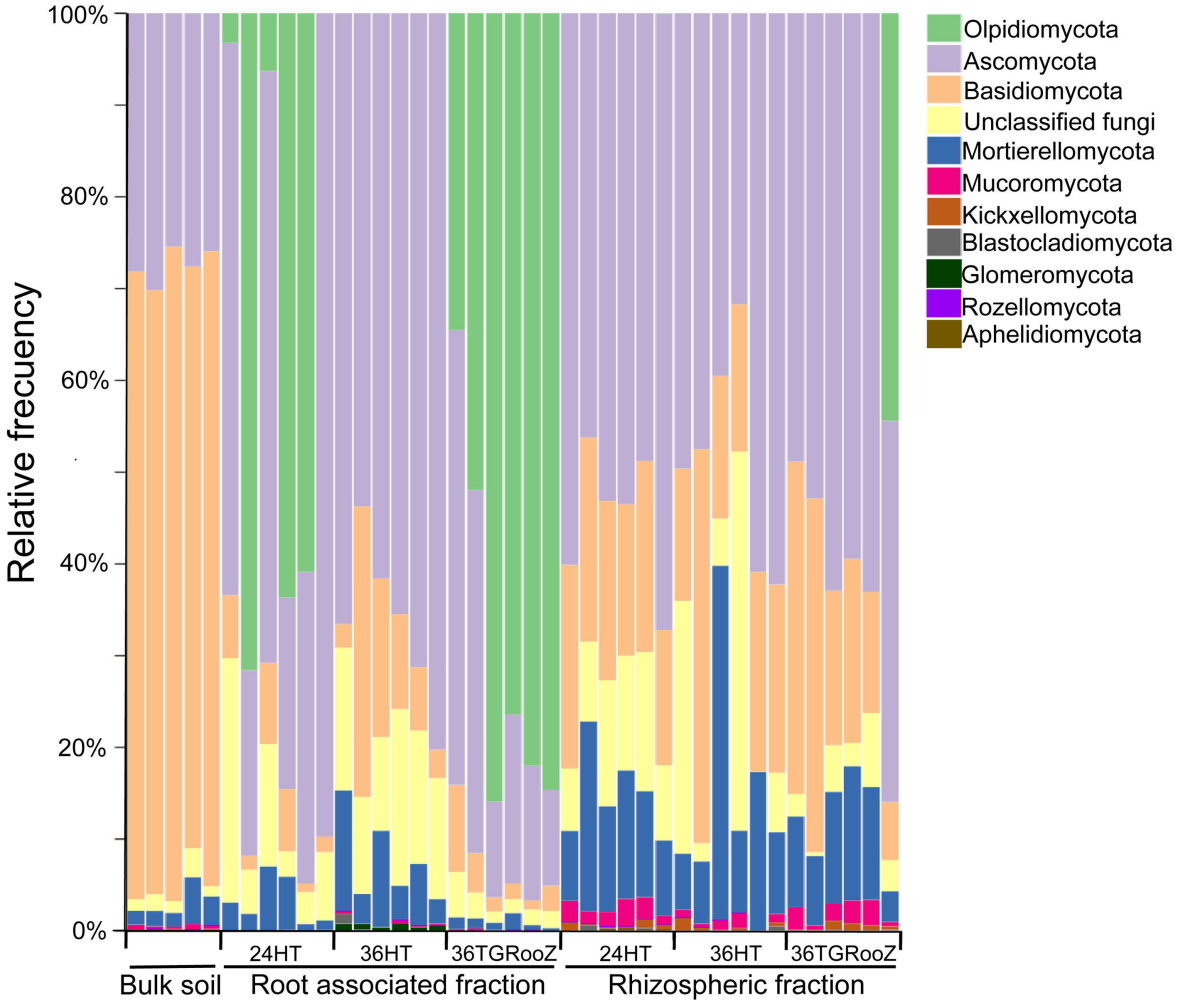
Taxa barplots. Taxonomic composition of bulk soil, rhizosphere and root-associated fraction of tomato plants grown at different temperatures: 24HT; 36HT, and 36TGRooz. Barplots represent the relative abundance of fungal taxa classified at phylum level. Note that *Glomeromycota*, *Rozellomycota*, and *Aphelidiomycota* represent less than 2% of all relative abundance in each fraction.

**Figure 3:**
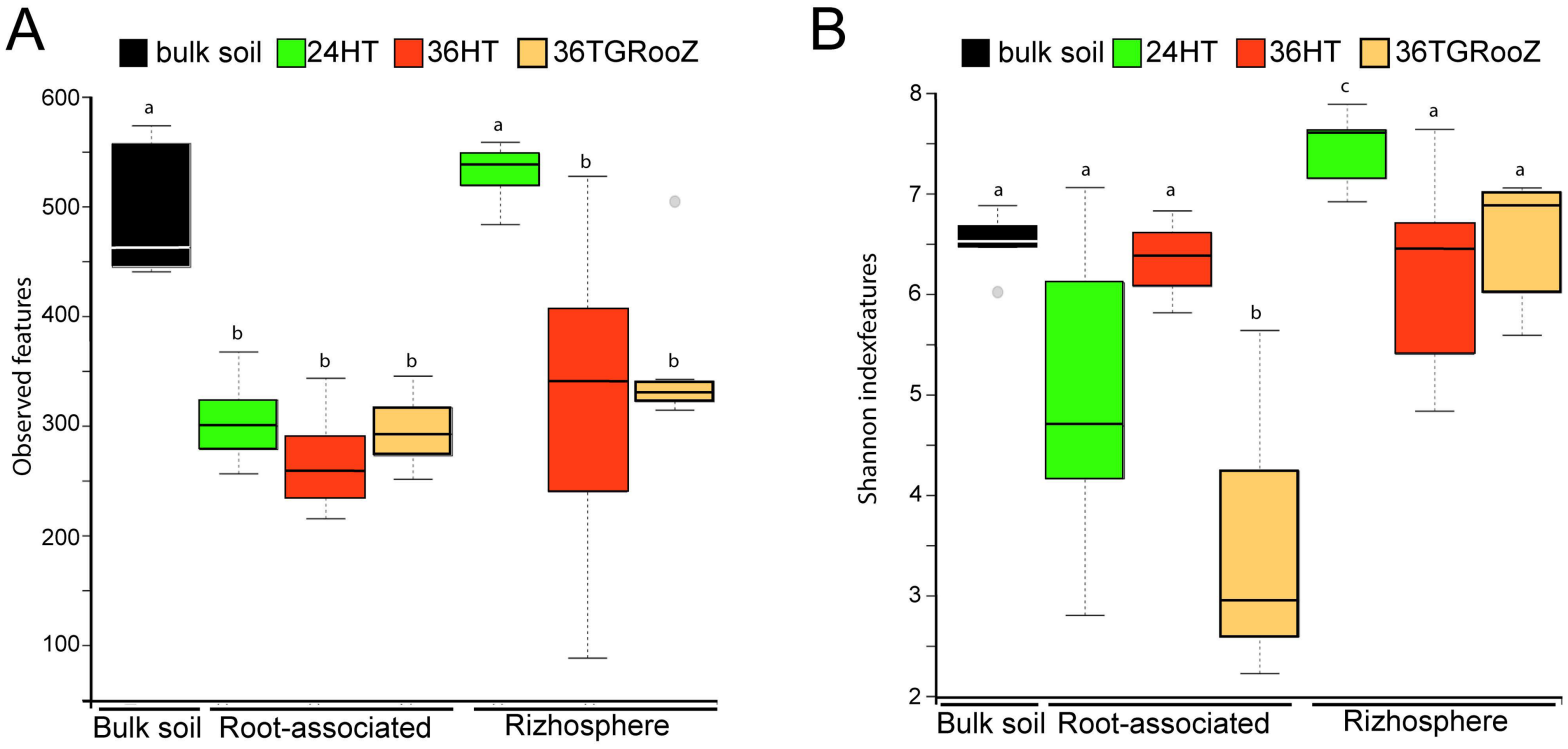
Effect of soil temperature on Alpha diversity. **(A)** Richness (observed features = observed ASVs). **(B)** Shannon index of bulk soil, rhizosphere and root-associated fractions of tomato plants grown at different temperatures (24°H, 24°C continuous, 36°H: 36°C continuous, 36TGRooZ: 36°C gradient). Internal line in box-plot boxes indicates the median or second quartile (Q2), upper line of the boxes indicates the third quartile (Q3) and lower line the first quartile (Q1) of the data. Different letters above the boxes indicate significant differences (*p* < 0.05) determined by non-parametric Kruskal-Wallis test.

Tomato plants were grown in three temperature conditions: at 24°C homogeneous in the shoot and root (24HT), at 36°C homogeneous in the shoot and root (36HT), and at 36°C in the shoot and with a temperature gradient from 36°C in the topsoil to 24°C in the deeper part of the pot (36TGRooZ). After 3 weeks, these different temperatures affected the shoot and root growth and the fresh and dried weight of the aerial part (Figure 1). Next, we isolated the DNA from the microbiota in the rhizosphere or root associated fractions, and the fungal community was analyzed by ITS amplicon sequencing and profiling. The fungal composition of each fraction was preliminarily obtained through the relative abundance of fungal taxa (Figure 2). ASVs taxonomically annotated to the phylum level showed that *Ascomycota* was the most abundant phylum across most samples, while other taxa showed variations in their relative abundance among samples depending on the fraction, temperature condition or both. Thus, *Basidiomycota* were less abundant in the rhizosphere fraction compared to bulk soil, while *Mortierellomycota* and *Mucoromycota* showed the opposite trend. No relevant differences were observed in terms of composition among the three different temperature treatments in bulk soil and rhizosphere fractions. However, in the root-associated fraction, *Olpidiomycota* was the most abundant phylum in samples grown at uniform 24°C or in 36TGRooZ, while it was virtually absent when roots were grown at uniform 36HT. Indeed, phyla composition of the root-associated fraction at uniform 36HT roughly resembled that of the rhizosphere fraction (Figures 2, 3), suggesting that this excessive high homogeneous temperature affects that correct root-microbiota assembly.

### 3.2 Diversity analysis

Fungal alpha diversity revealed differences in richness and Shannon index across samples, depending on the fraction and temperature condition (Figure 3). Regarding fungal richness, measured as the number of observed ASVs (Figure 3A), both bulk soil and rhizosphere fractions of plants grown at 24HT exhibited the highest richness, with approximately 500 observed ASVs. These values were significantly higher than those of the other samples (*p* < 0.05). No significant differences were found between rhizosphere fractions of plants grown at 36HT and 36TGRooz, nor among the root-associated fractions across the three temperature treatments, with a median number of observed ASVs ranging between 250 and 350. Notably, the rhizospheric sample grown at 36°C displayed a higher degree of variability.

**Figure 4:**
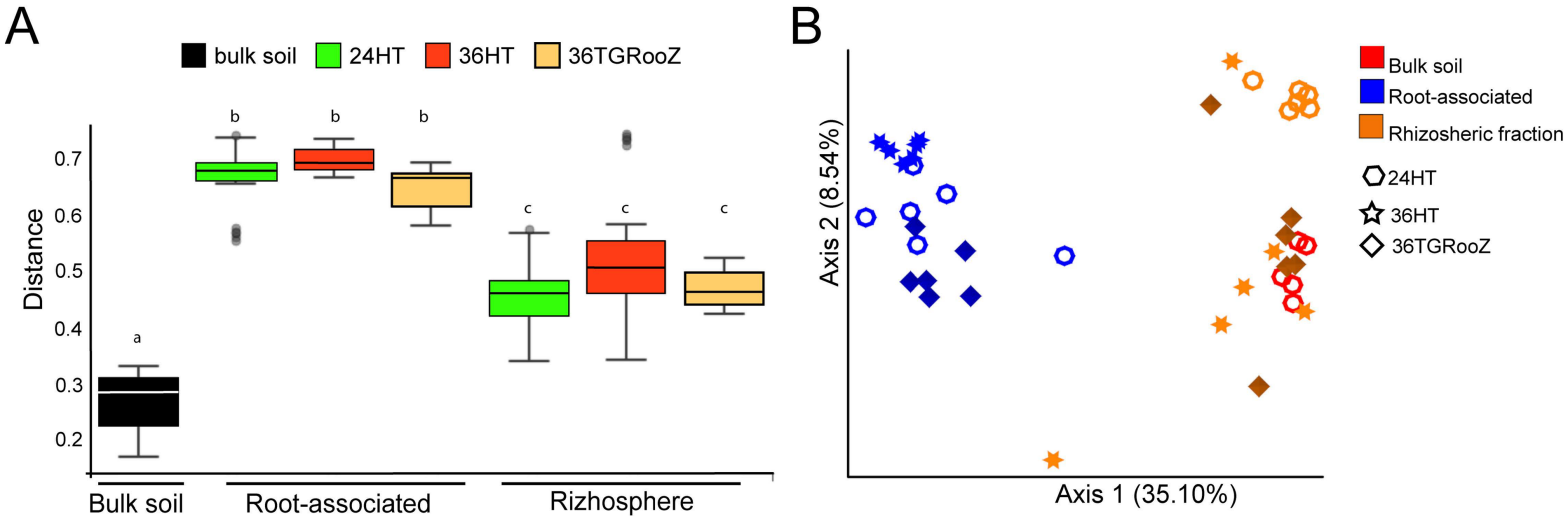
Beta diversity of bulk soil, rhizosphere and root-associated fractions of tomato plants grown at different temperatures. **(A)** UniFrac distance boxplots relative to unplanted bulk soil. Internal line in box-plot boxes indicates the median or second quartile (Q2), upper line of the boxes indicates the third quartile (Q3) and lower line the first quartile (Q1) of the data. **(B)** Principal coordinate analysis (PCoA) based on unweighted UniFrac distance of the fungal communities unconstrained by fraction (bulk soil, rhizosphere and root-associated) and temperature condition (24°C homogeneous, 36°C homogeneous, 36°C TGRooZ gradient) (*n* = 41).

Shannon index (Figure 3B), also varied significantly among temperature conditions. Within the rhizospheric fraction, plants grown at 24HT showed a significantly higher diversity, with an average index of 7.6 (*p* < 0.05), being even significantly higher than the one of the bulk soil. In the case of the root-associated fraction, plants grown at 36TGRooZ condition exhibited a significantly lower median Shannon index (2.95, *p* < 0.05) than those grown at 24HT or 36HT. Interestingly, Shannon index of root-associated fraction of plants grown at 36HT was similar to those of the rhizosphere fractions and the bulk soil. The analysis of fungal beta diversity (Figure 4) was performed by measuring unweighted UniFrac distances. PERMANOVA analysis showed that the fraction was the main driver of variation among samples (*F* = 7.10, *p* < 0.01), UniFrac distances using bulk soil as reference shows differences between fractions: bulk soil, rhizosphere fraction, and root-associated fraction (Figure 4A). UniFrac distances among samples were used to build a PCoA plot (Figure 4B), which revealed two distinct clusters along the first axis, separating the root-associated samples from the soil-related fractions (bulk soil and rhizosphere). The effect of temperature appeared along the second axis, showing a gradual separation of root-associated samples grown at 36HT and 36TGRooZ conditions, with samples grown at 24HT located in between. Rhizosphere and bulk soil samples also tended to group by temperature condition, with rhizosphere samples grown at 24°C showing a clear distinction from the rest. Given that the fungal community composition was markedly different between the rhizosphere and root-associated fractions, it is likely that subtle variations due to temperature were masked in the combined analysis. Therefore, to specifically assess the effect of temperature, beta diversity analyses were subsequently performed separately for root-associated and rhizosphere samples, excluding bulk soil.

**Figure 5:**
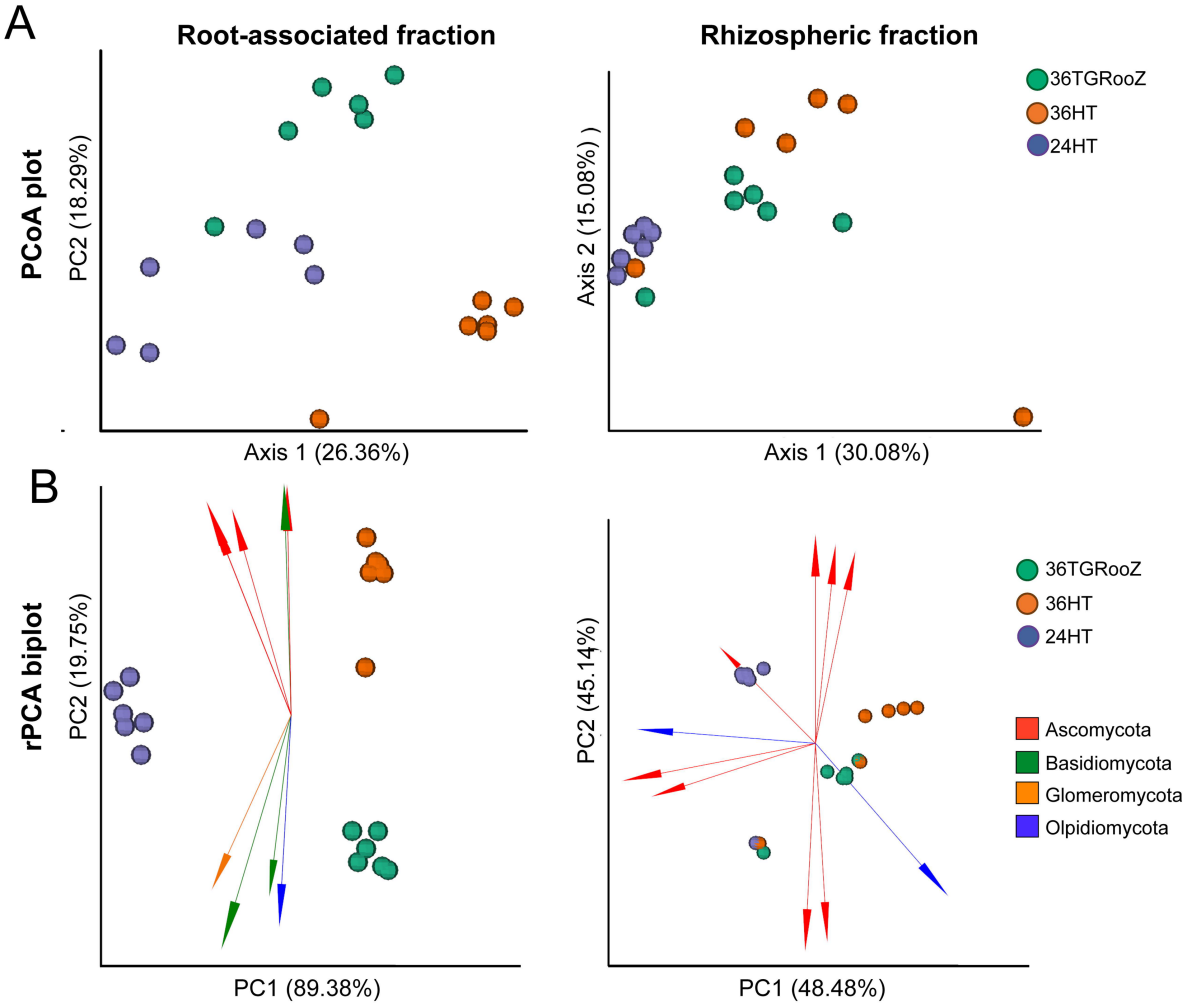
Beta diversity of the rhizosphere and root-associated fractions. **(A)** Principal coordinate analysis (PCoA) based on UniFrac distance of fungal communities of the rhizosphere (left) and root-associated (right) fractions unconstrained by temperature condition (24°C homogeneous, 36TGRoot gradient, 36°C homogeneous) (*n* = 18). **(B)** Robust principal component analysis (rPCA) based on Aitchison distance of fungal communities of the root-associated (left) and rhizosphere (right) fractions. Arrows represent the loading plots of the ten main ASVs driving variability in each fraction.

Independent beta diversity analysis of each fraction, measured by unweighted UniFrac distance, and PERMANOVA analyses revealed a significant effect of temperature condition in both fractions (Rhizosphere: *F* = 3.1; *p* < 0.01; Root associated: *F* = 5.1; *p* < 0.01). PCoA plots showed that the rhizosphere samples of plants grown at uniform 24°C grouped separately from the rest of the samples and samples from the root-associated fraction of plants grown at uniform 36°C were also distinctly separated from the rest of the root-associated samples (Figure 5A). The effect of the temperature condition on rhizosphere and root-associated fractions was further analyzed using robust PCA (rPCA) based on Aitchison distance (Figure 5B). Confirming a significant effect of temperature for both fractions (PERMANOVA Rhizosphere *F* = 7.8; *p* < 0.01; Root associated *F* = 286; *p* < 0.001). The rPCA plots of the rhizosphere fraction agreed with UniFrac distance analyses and confirmed that samples grown at 24°C clearly grouped together, while the rest of the samples formed a more diffuse group that could not be separated into distinct temperature conditions. In this case, the first two components explained 93% of the total variance, each contributing similarly. In contrast, the rPCA of the root-associated fraction showed that the three temperature conditions were clearly separated into distinct clusters. Here, 89% of the total variance was explained by the first component, which separated the samples grown at 24HT (on the left) from the clusters corresponding to 36HT (top-right) and 36TGRooZ (bottom-right).

rPCA plots were also used to represent the ten main ASVs driving variability in a compositional biplot. In the rhizosphere fraction, most of the loading plots (arrows) of these ten ASVs appeared scattered in multiple directions, with no clear association to any cluster. Most of these ASVs were classified within the phylum *Ascomycota*, except for two ASVs belonging to *Olpidiomycota*. One ASV from Ascomycota seemed to be associated with the rhizosphere fraction of plants grown at uniform 24HT, while an *Olpididiomycota* ASV was linked to the cluster mainly by 36TGRooZ samples. In contrast, rPCA biplot of the root-associated fraction showed a clear directional trend in the loading plots (arrows) of the ten main ASVs along the second axis, which separated the clusters of samples grown at 36HT (top) from the cluster of 36TGRooZ samples (bottom of the plot). Taxonomic metadata associated with these driving ASVs revealed that those pointing upwards, in the direction of 36HT samples were classified in the phyla *Ascomycota* and *Basidiomycota*, whereas the arrows pointing downwards, in the direction of 36TGRooZ belonged to the phyla Glomeromycota, Basidiomycota, and Olpidiomycota (Figure 5B).

The beta diversity results suggest that the effect of temperature condition was more pronounced in the root-associated fraction than in the rhizosphere. Therefore, we focused subsequent analyses on investigating how different temperature conditions influenced the assembly of the fungal community associated specifically with tomato roots, through differential abundance analysis.

### 3.3 Taxonomic distribution of root-associated ASVs across samples

**Figure 6:**
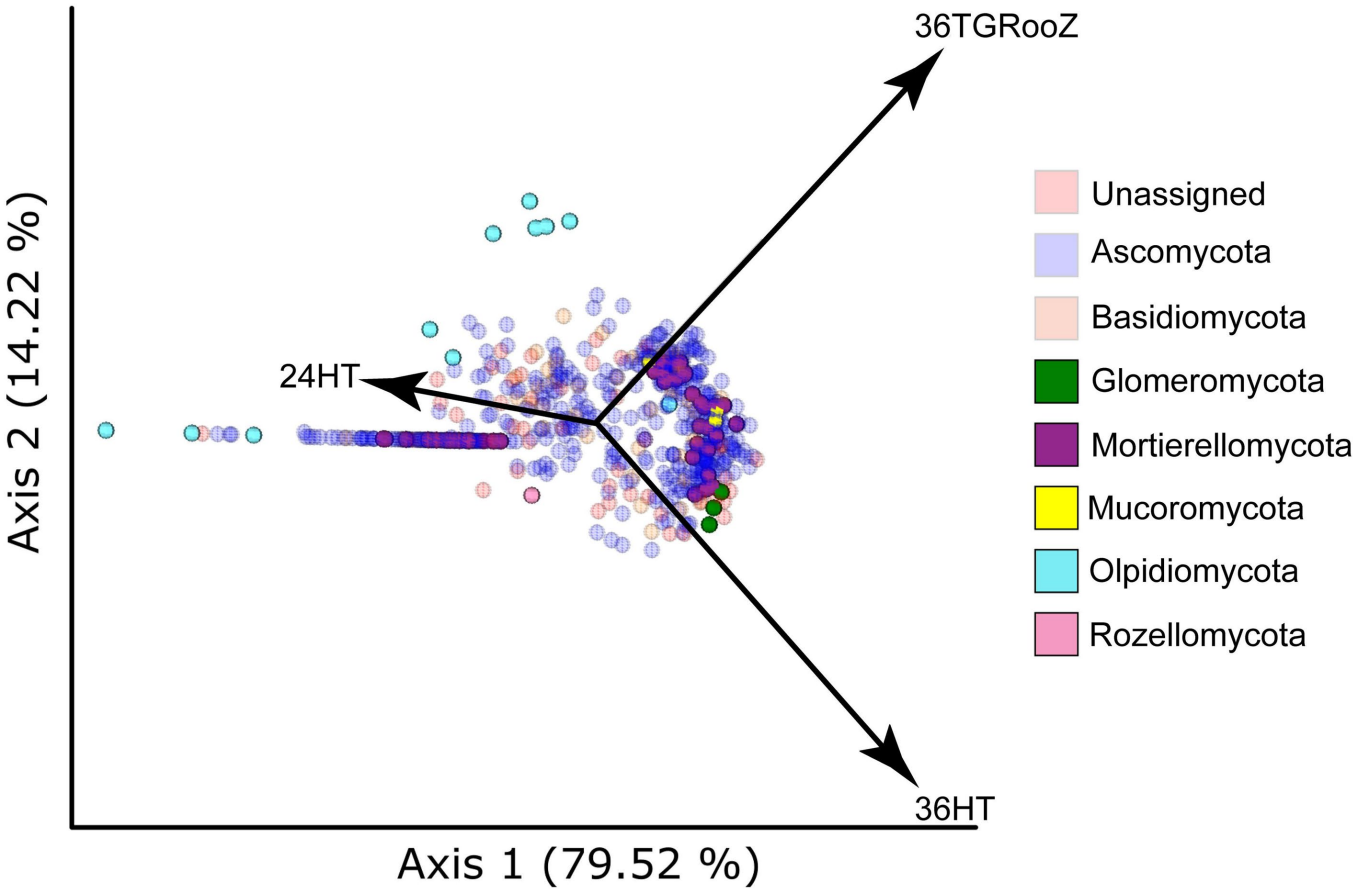
Principal Coordinate Analysis (PCA) plot based on SONGBIRD differentials. The plot shows the distribution of ASVs in the root-associated fraction according to temperature conditions. Dots represent individual ASVs colored by its taxonomy at the phylum level. Taxa shown in a dimmed color in the legend are also dimmed in the plot. Arrows indicate the temperature condition (24HT: 24°C continuous, 36HT: 36°C continuous, 36TGRooz: 36°C gradient).

Samples taken from the root-associated fraction were analyzed using SONGBIRD to find how the ASVs were clustered according to their relative abundance across the three temperature conditions (Figure 6). After dimming the ASVs classified in the phyla *Ascomycota* and *Basidiomycota*, whose abundance masks the underlying differences, four phyla appeared with a significant abundance in the biplot: *Olpidiomycota*, *Mucoromycota*, *Glomeromycota*, and *Mortierellomycota*. Several ASVs classified in the phylum *Olpidiomycota* were associated to 24HT while some others were distributed between 24HT and 36TGRooZ conditions. ASVs classified in the phylum *Mucoromycota* tended to cluster between the conditions 36HT and 36TGRooZ but showed no association to 24HT. Three ASVs of the phylum *Glomeromycota* were clearly linked to 36HT, while ASVs of the phylum *Mortierellomycota* were distributed in two clusters, one associated to 24HTand the other one between 36HT and 36TGRooZ.

### 3.4 Analysis of differentially abundant ASVs in the root-associated fraction

**Figure 7:**
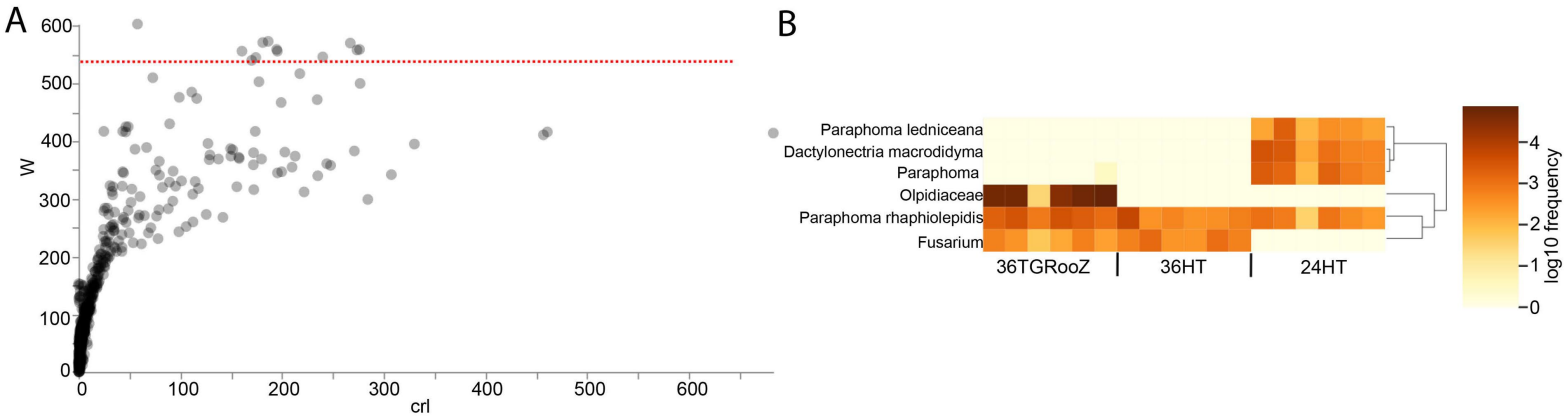
Analysis of Compositions of Microbiomes (ANCOM) in the root-associated fraction. **(A)** Differentially abundant ASVs identified by ANCOM. Those above the threshold line (red line), established by the W-statistic, are considered significant. **(B)** Heatmap of significantly differentially abundant ASVs grouped by taxonomy.

**Figure 8:**
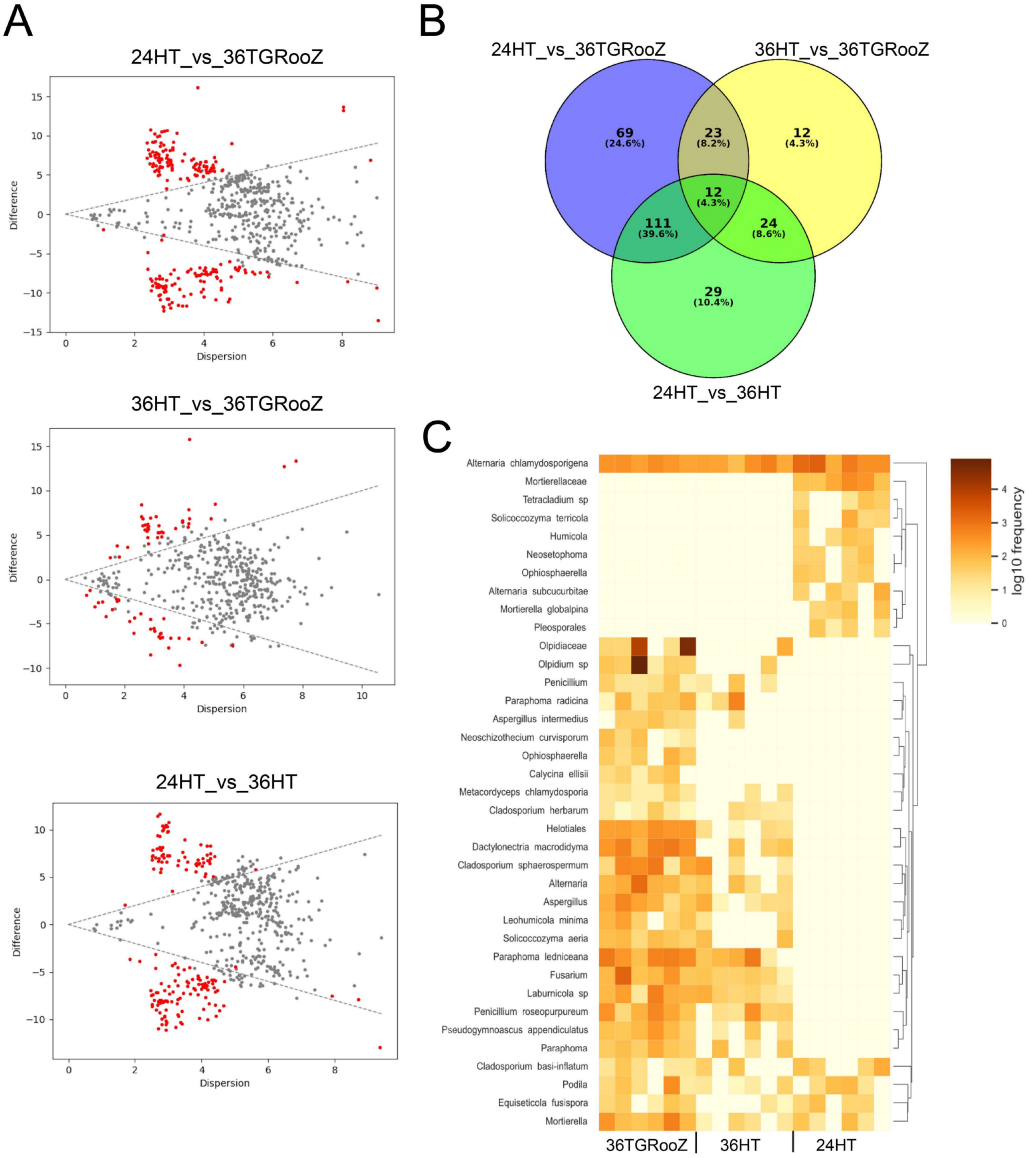
Pairwise analysis based on ANOVA-Like Differential Expression (ALDEx2) of the fungal community of the root-associated fraction. **(A)** Effect plot of each pairwise temperature comparison. Differentially abundant ASVs are represented by red dots. **(B)** Venn diagram of differentially abundant ASVs across temperature conditions. **(C)** Heatmap of the 69 ASV differentially abundant in 36TGRoooZ compared to 24HT grouped in 37 taxa.

To go deeper into the fungal microbiome gathered in tomato roots in response to the different temperature conditions, their differential composition was studied using both overall analysis and pairwise comparisons. For that, two different methods were used: ANCOM (Figure 7) and ALDEx2 (Figure 8). It has already been published that ANCOM-II and ALDEx2 are one of the most robust approaches to analyze differentially abundant taxa in the composition of microbiome communities (Nearing et al., 2022). This work uses ANCOM as a proxy to ANCOM-II to perform the analysis of fungal communities across all temperature conditions, and ALDEx2 to study the differential composition of the fungal communities in pairwise comparisons.

The output of ANCOM analysis was filtered according to the taxonomic metadata to plot their relative abundance in all the temperature conditions. Twelve ASVs classified into six different taxa showed a significant differential abundance across the three temperature conditions (Figure 7A), as seen in the heatmap (Figure 7B). Three of these taxa could be identified at the species level: *Paraphoma ledniceana* and *Dactylonectria macrodidyma*, which are only present at 24HT, and *Paraphoma rhaphiolepidis*, whose abundance was stable across all three temperature conditions. The ASVs of the family Olpidiaceae were enriched in the 36TGRooZ condition, while the ASVs of genus *Fusarium* were depleted at 24HT.

ANCOM is known to be a very conservative analysis, yielding a low number of differentially abundant taxa. Therefore, the root-associated fraction was further analyzed with ALDEx2 using a pairwise approach to find enriched or depleted taxa in each temperature condition. Effect plots were obtained for the three possible pairwise comparisons among the conditions 24HT, 36HT and 36TGRooZ (Figure 8A) and the lists of differentially abundant ASVs were used to create a Venn diagram (Figure 8B). Sixty-nine ASVs were differentially abundant in 36TGRooZ compared to the standard conditions 24HT (Supplementary Table S1.1), which were classified into 37 different taxa (Supplementary Table S1.2), with 19 of them classified at species level. The abundance of these taxa was plotted in a heatmap (Figure 8C) that showed two main clusters. Cluster 1 was formed by 9 taxa present at 24HT and practically absent in the other two conditions. The species *Alternaria chlamydosporigena* grouped apart because, although enriched at 24HT, was also present in the other 2 conditions. Cluster 2 grouped taxa enriched in 36TGRooZ. This was the biggest group, with 23 taxa, and included members of many different families and genera like *Fusarium, Aspergillus, Cladosporium* or *Alternaria*. In this cluster, only ASV of taxa *Schizothecium curvisporum*, *Ophiosphaerella* sp. and *Calycina ellisii* are practically exclusive of 36TGRooz. All other taxa appear also, although less abundantly, at 36HT, with 4 of them forming a subcluster because they are also present at 24HT.

## 4 Discussion

The effect of high temperatures on plants has become a critical field of study due to the increasing threats posed by climate change (Zhu et al., 2021). Understanding how plants cope with heat stress is essential for developing strategies to mitigate its negative impact. Most current experimental setup in controlled environments (chambers o greenhouses) have exposed both roots and aerial parts of plants to uniformly high temperatures (Hatfield and Prueger, 2015; Giri et al., 2017). However, in nature, the soil acts as a thermal buffer, with deeper layers less affected by surface temperature fluctuations, creating a vertical gradient from warmer surface to cooler depths. This gradient is essential to protect the root system and its associated microbiome from excessive high temperatures (González-García et al., 2023). Our previous work demonstrated that applying a temperature gradient using the TGRooZ device, either in pots or plates, produces a more realistic environment and is better suited for studying the genetic and cellular mechanisms underlying plant responses to heat (González-García et al., 2023). We also found that soil temperature strongly influences the root-associated bacterial microbiome dynamics, with dramatic shifts observed under constant high temperatures. In this study, we extend our analysis to fungi, another key component of the soil-root microbiome. Fungi and bacteria can respond differently to environmental factors such as humidity, soil composition, and temperature (Fierer, 2017; Li et al., 2023; Díaz-González et al., 2025). Heat stress primarily elevates the temperature of the aerial parts of the plant, inducing specific physiological responses. In contrast, the root zone and associated microbiome typically experience a more moderate temperature due to the formed gradient. Therefore, accurately assessing the responses of the plant, root system, and soil microbiome to heat stress requires a carefully designed experimental setup that reflects these spatial temperature differences.

Our results using the TGRooZ system demonstrate that elevated temperatures in the root growth zone significantly influence the assembly of fungal communities. This effect is likely driven by the abnormally high and uniform temperatures applied in conventional homogeneous setups. These findings further underscore the importance of naturalistic experimental designs, such as incorporating soil temperature gradients, for generating more accurate and ecologically relevant conclusions.

### 4.1 Fungal diversity depends largely on the root compartment

The preliminary taxonomic analysis of the data (Figure 2) shows that fungal composition at the phylum level is mostly affected by the type of sample, plant or soil associated (bulk and rhizosphere), with temperature conditions affecting each fraction differently. This finding is consistent with previous studies supporting the widely accepted conclusion that the plant’s influence on the microbiome is generally stronger than that of other analyzed environmental factors (Edwards et al., 2015; Lee et al., 2019; Díaz-González et al., 2025). The rhizosphere fraction shows, as a general trend, an enrichment of specific ASVs classified in the phylum *Mortierellomycota* and *Mucoromycota*, when compared to the bulk soil. This enrichment happens irrespective of the temperature condition (Figure 2). However, in the case of the root-associated fraction, the fungal composition is clearly different when plants have been grown at different temperatures. For example, the samples of 24HT and 36TGRooZ roots (cooler environments) are enriched in ASVs classified in the phylum *Olpidiomycota*, which are quite common in association with the roots of tomato (Anzalone et al., 2022). However, this phylum is almost absent from samples grown at uniform 36HT. It has been shown that some arbuscular mycorrhizal fungi show impaired colonization of their host’s roots in high temperature (Martin and Stutz, 2004), suggesting that *Olpidiomycota* fungi might be sensitive to heat as well. Conversely, the root-associated samples grown at 36°C show an enrichment in fungi from phylum *Glomeromycota*, whose members are known to colonize roots and form arbuscular mycorrhizae (Brundrett and Tedersoo, 2018). Whether this enrichment is the result of an adaptive recruitment of beneficial phyla by tomato roots due to hostile environmental conditions, or it is a sign of fitness loss due to extreme heat need further studies.

It is well established that plant roots exert a strong selective pressure on microbial communities, often leading to a reduction in alpha diversity (Díaz-González et al., 2025; Zhang et al., 2022). This phenomenon likely accounts for the lower diversity observed in root-associated samples under the 24HT condition compared to the rhizosphere. In contrast, the reduced diversity detected in rhizosphere samples maintained at 36HT, as well as in the 36TGRooZ condition, suggests that elevated temperature exerts an independent selective influence. Interestingly, no significant effect of temperature on the alpha diversity of root-associated fungal communities was observed. This may be due to the dominant selective role of the plant, which could override or obscure the effects of temperature, an interpretation consistent with findings from studies examining other environmental variables (Edwards et al., 2015; Lee et al., 2019; Díaz-González et al., 2025).

Independent analysis of beta diversity in the rhizosphere and root-associated fractions reveals that temperature condition influences the assembly of fungal communities selected by tomato roots, showing that rhizosphere samples of plants grown at 24HT form a separate cluster, while within the root-associated fraction, samples from plants grown at 36HT stand out as the most distinct. These results differ somewhat from those reported for bacterial communities by González-García et al. (2023), where the 36HT condition stood out in both rhizosphere and root associated samples. Nonetheless, they are consistent with the overall trends observed in the alpha and beta diversity analyses of fungal communities across all root fractions and temperature conditions, as well as with the general taxonomic distribution at the phylum level.

UniFrac distance accounts for phylogenetic relationships, while Aitchison distance captures relative abundance patterns, making it more suitable for sparse metagenomic data (Armstrong et al., 2022). rPCA biplots further highlight key features driving sample separation. Notably, samples from plants grown at 24HT form a distinct cluster, separate from those at 36HT, regardless of root temperature—suggesting shoot-to-root signaling under heat stress (Vescio et al., 2021; González-García et al., 2023). Root-associated samples also cluster by soil temperature, indicating additional regulation via root-localized heat perception or stress damage (Nottingham et al., 2019).

Taken together, our data indicate that root proximity is a stronger driver of fungal community differentiation than temperature alone, although soil temperature clearly influences rhizosphere fungal composition. Consequently, the use of uniformly elevated temperatures in the root zone may introduce experimental bias and lead to conflicting or misleading results.

### 4.2 The temperature gradient affects the composition of root associated fungal communities

Changes in the fungal community associated with tomato roots under different temperature conditions appear to be primarily driven by heat perception in the aerial parts of the plant. In the case of root-associated samples, the fungal communities can be further distinguished between those exposed to 36HT and those under the 36TGRooZ condition. Given that TGRooZ mimics the conditions found in nature, the main goal of this work is to compare the conventional laboratory condition of 24HT with 36TGRooZ in order to investigate the responses of root fungal communities of plants growing under high atmospheric temperature. ALDEx2 pairwise comparisons yielded 69 differentially ASVs from which only 19 taxa could be classified at the species level. This subset was used for a literature review to explore the potential roles of these species in plant growth, yield or thermotolerance.

Several fungal species enriched under the 36TGRooZ condition are known to benefit plants by promoting growth or offering protection against pathogens. For instance, *Cladosporium* *sphaerospermum* enhances tomato yield via gibberellin pathway modulation (Hamayun et al., 2009, 2010; Li et al., 2018), while *Solicoccozyma aeria* produces IAA and triggers auxin responses in maize and Arabidopsis, both *in vitro* and in natural soils (Ramos-Garza et al., 2023; Carvajal et al., 2023). It has also been shown to promote growth in non-host crops. Additionally, *Metacordyceps chlamydosporia* acts as a biocontrol agent by parasitizing *Meloidogyne eggs*, protecting tomato roots from nematode infestation (Ghahremani et al., 2019; Hajji-Hedfi et al., 2022). Many of the differentially enriched species under the 36TGRooZ condition belong to taxonomic groups known to include both pathogenic and beneficial fungi (Rodriguez and Redman, 2008), and some taxa are even capable of switching lifestyles depending on different conditions. For example, *Paraphoma radicina* has traditionally been regarded as a pathogen (Dang and Li, 2022), while some strains of this species have been shown to provide protection against abiotic stress to non-host crops (Li et al., 2022). Another example is *Cladosporium herbarum*. Although known as pathogen of *Passiflora* crops, it produces exudates containing glucosylceramides that have been shown to trigger the plant immune response and promote plant growth (Xisto et al., 2022). Further work would be necessary to ascertain if the presence of these fungi has a detrimental effect or really contributes to improving plant performance under high temperature conditions.

Some fungi enriched in the root-associated fraction under 36TGRooZ may be selected for their thermotolerance or resilience to heat-associated stresses like salinity and drought (Duc et al., 2018). For example, *Leohumicola minima* belongs to a thermotolerant genus (Hambleton et al., 2005), while *Cladosporium sphaerospermum* and *Penicillium roseopurpureum* are known for halo-and osmotolerance (Zalar et al., 2007; Min et al., 2014). Conversely, taxa such as *Alternaria* spp.—common tomato pathogens—were less abundant under 36TGRooZ. This reduction may reflect lower stress tolerance, plant-mediated suppression, or competitive exclusion by beneficial fungi.

In summary, tomato roots grown under 36TGRooZ conditions host a distinct fungal community compared to those exposed to uniform high temperatures. While the role of this community in enhancing thermotolerance remains to be confirmed, our findings highlight the importance of soil temperature gradients in shaping beneficial root-associated microbiomes. This study also emphasizes the complex ecological roles of these fungi and the need to further investigate their contributions to plant resilience. Additionally, the differential fungal assembly suggests a shoot-to-root signaling mechanism, where heat perception in aerial tissues influences root microbiota, a process previously observed under uniform conditions (Chaparro et al., 2014; Kim et al., 2022), but here shown in a more field-realistic context (González-García et al., 2023).

## Data Availability

The original contributions presented in the study are publicly available in the SRA repository. The original data can be found here: https://www.ncbi.nlm.nih.gov/sra/SRX22565450[accn], number: PRJNA1041460].

## Footnotes

^1^ https://biomemakers.com/

^2^ https://github.com/pczoido/AUTOTRUNC.git
